# Imaging Cardiovascular Inflammation in the COVID-19 Era

**DOI:** 10.3390/diagnostics11061114

**Published:** 2021-06-18

**Authors:** Andras Mester, Imre Benedek, Nora Rat, Cosmin Tolescu, Stefania Alexandra Polexa, Theodora Benedek

**Affiliations:** 1 Department of Cardiology, George Emil Palade University of Medicine, Pharmacy, Science and Technology of Targu Mures, 540142 Târgu Mureș, Romania; andras.mester@yahoo.com (A.M.); imrebenedek@yahoo.com (I.B.); theodora.benedek@gmail.com (T.B.); 2 Cardiology Clinic of the Emergency Clinical County Hospital of Târgu Mureș, 540142 Târgu Mureș, Romania; tolescu.cosmin@yahoo.com (C.T.); polexa.stefania@gmail.com (S.A.P.)

**Keywords:** COVID-19, cardiac magnetic resonance, inflammation, multimodal imaging, myocardial injury, artificial intelligence

## Abstract

Cardiac complications are among the most frequent extrapulmonary manifestations of COVID-19 and are associated with high mortality rates. Moreover, positive SARS-CoV-2 patients with underlying cardiovascular disease are more likely to require intensive care and are at higher risk of death. The underlying mechanism for myocardial injury is multifaceted, in which the severe inflammatory response causes myocardial inflammation, coronary plaque destabilization, acute thrombotic events, and ischemia. Cardiac magnetic resonance (CMR) imaging is the non-invasive method of choice for identifying myocardial injury, and it is able to differentiate between underlying causes in various and often challenging clinical scenarios. Multimodal imaging protocols that incorporate CMR and computed tomography provide a complex evaluation for both respiratory and cardiovascular complications of SARS-CoV2 infection. This, in relation to biological evaluation of systemic inflammation, can guide appropriate therapeutic management in every stage of the disease. The use of artificial intelligence can further improve the diagnostic accuracy of these imaging techniques, thus enabling risk stratification and evaluation of prognosis. The present manuscript aims to review the current knowledge on the possible modalities for imaging COVID-related myocardial inflammation or post-COVID coronary inflammation and atherosclerosis.

## 1. Introduction

The coronavirus disease 2019 (COVID-19) outbreak laid an unparalleled burden on healthcare systems worldwide. The disease caused by the severe acute respiratory syndrome coronavirus-2 (SARS-CoV-2) mainly affects the respiratory system, manifesting from atypical forms of pneumonia to fatal acute respiratory distress syndrome (ARDS). The emerging literature data revealed the fact the extrapulmonary manifestations of the viral infection have an important impact in the development of severe forms, being associated with increased mortality rates [1]. The first published data have shown worse outcomes, higher incidence of complications and requirement of intensive care in patients with underlying cardiovascular disease and also a five-to-tenfold increase in mortality rates [2]. Patients with a known history of cardiovascular disease also exhibited higher mortality rates, irrespective of the cause of hospitalization (respiratory or cardiac symptoms); however, multivariate analysis evidenced that death was mainly associated with older age and respiratory distress [3,4]. The exact pathophysiological mechanism of cardiovascular involvement is still not well elucidated. Numerous early studies have reported signs of myocardial injury in approximately one third of COVID-19 patients, expressed by imaging methods (echocardiography or cardiac magnetic resonance imaging) or marked elevations of cardiac biomarkers such as troponin T. The elevation of troponin T levels was also associated with higher complication rates and lower short-term survival [5,6,7]. In addition, plasma high sensitivity troponin T levels were identified as independent predictors for high mortality rates [4].

One actual hypothesis is that enhanced systemic inflammatory response directly affects the myocardial cells, leading to local inflammation and myocardial injury. At the same time, it may trigger a local reaction within coronary plaques, leading to rupture, thrombosis and subsequent acute coronary events [8]. In addition, inflammation is also associated with an increased propensity for thrombosis in the microcirculation, which is similar to the pathogenesis of disseminated intravascular coagulation (DIC). The over-activation of the immunological system as a response to the SARS-CoV-2 infection may have deleterious effects, triggering the cytokine storm with subsequent tissue injury and organ damage [9]. It has been recently postulated that the persistence of inflammation after complete recovery will contribute to development and progression of left ventricular dysfunction and subsequent heart failure [10,11].

Echocardiography represents the first line imaging method for assessing cardiac function, and it is essential in referring patients to second level imaging techniques; however, its use has been limited during the pandemic given the high exposure risk of the operators and danger of spreading the viral particles during the direct contact of the patients with the equipment. Cardiac magnetic resonance imaging (CMR) is the method of choice for imaging inflammation, edema or diffuse myocardial fibrosis, while multi-slice computed tomography angiography (CTA) is the imaging method of choice for visualization of coronary plaques and assessment of plaque vulnerability. Therefore, in the context of COVID-19 pandemic, cardiac magnetic resonance should be the method of choice for investigating myocardial involvement while CTA should be used to assess coronary atherosclerosis and inflammation-mediated coronary plaque vulnerabilization. However, despite their relevant contribution to diagnose cardiac involvement, COVID complications or post-COVID cardiovascular risk, these are still underused diagnostic tools.

The present manuscript aims to review the current knowledge on the possible modalities for imaging COVID-related myocardial inflammation or post-COVID coronary inflammation and atherosclerosis.

## 2. Inflammation and Cardiovascular Diseases

The role of an increased systemic inflammatory status in the development of atherosclerotic plaques and their progression to a vulnerable phenotype leading to acute coronary syndromes (ACS) has been extensively studied in the recent years. The usefulness of inflammatory biomarkers such as high sensitive C- reactive protein (hs-CRP) has been demonstrated in numerous clinical trials, being currently considered an independent predictor for the mortality rate of major cardiovascular events [12,13]. Cytokines are also emerging serum biomarkers for assessment of systemic inflammation. Higher levels of interleukins (IL-6, -18) and tumor necrosis factor alpha (TNF-α) were also associated with development of coronary artery disease (CAD), cerebrovascular diseases and ACS, but their exact role is yet to be determined in larger clinical trials [14,15,16].

A chronic systemic inflammatory status has been associated with atherosclerotic plaque formation and, furthermore, with triggering acute coronary events [17]. Autoimmune diseases such as lupus erythematosus, psoriasis and rheumatoid arthritis are associated with increased rates of cardiovascular events at lower ages compared to the age-gender matched group without these conditions [18].

Alongside the systemic inflammatory state, local vascular inflammation promotes endothelial dysfunction, plaque formation and destabilization and ultimately leads to acute coronary events. The perivascular adipose tissue (PVAT) plays an important role in local plaque inflammation by paracrine signaling via pro-inflammatory cytokines (IL-6, TNF-α) and adhesion molecules (e.g., vascular cell adhesion molecule-1, intercellular adhesion molecule-1, E-selectin, P-selectin) [19,20]. Recent CT and CMR studies emphasize the role of PVAT as a new possible imaging biomarker for prediction of cardiovascular events [21].

## 3. Cytokine Storm and Cardiac Injury

Current data show that 20–40% of COVID-19 patients present clinical signs or laboratory biomarkers of myocardial injury (high sensitive cardiac troponin, creatine kinase-MB), predominantly in those who are treated in intensive care units with an associated poorer prognosis [22]. The cardiac involvement in SARS-CoV-2 infection generates a different range of manifestations (acute coronary syndromes: STE and non -STE, heart failure, arrhythmias, sudden cardiac death, myocarditis, pericarditis, Takotsubo syndrome, pulmonary embolism) caused by direct myocardial damage, cytokine storm, thrombosis, endothelial dysfunction and impaired metabolic activity. Following the anchoring of SARS-CoV-2 spike protein to the angiotensin converting enzyme 2 (ACE2) receptor (expressed in the respiratory-, cardiovascular-, immune-, gastrointestinal-, central nervous system, kidney and liver), the virus migrates into the target cell and begins the replication process, leading to cell damage or destruction and the activation of the immune system [9,23,24]. ACE2 plays an important role in mediating the immune response by directly interacting with macrophages and reducing the pro-inflammatory, pro-athero-thrombotic and oxidative effect of angiotensin II (AT II) [25,26]. In SARS-CoV-2 infection, a down-regulation of the surface ACE2 receptors (as a possible protective mechanism against the viral invasion) was observed with subsequent elevation of circulating AT II and TNF-α levels, leading to increased fibrosis, saline retention and marked vasoconstriction. Moreover, there is a reduction of cardioprotective AT 1–7 levels [27,28,29]. In patients with severe forms of COVID-19, an overactivation of the immune system and decrease of mediator (such as ACE2) activity causes excessive pro-inflammatory cytokine release (e.g. IL-6, -2, -10, -22, -1β, TNF-α, interferon γ, granulocyte-colony stimulating factor), known as the “cytokine storm” which leads to a deleterious local and systemic inflammatory response and subsequent multiple organ failure [30,31]. The emerging literature data from recently published clinical trials on severe COVID-19 patients revealed that high circulating levels of IL-6, IL-1β, IL-8 and TNF-α were associated with poor prognosis and higher mortality rates [32,33,34]. Furthermore, IL-6 levels were correlated with the viral load and disease progression, evidenced by CT in severe COVID-19 patients and was proposed as a possible prognostic biomarker for the forthcoming cytokine storm and severe disease [35,36,37].

The exact pathophysiological mechanism of cardiac injury in COVID-19 patients is still under debate. Direct myocardial damage due to viral involvement and replications has been evidenced. In an autopsy study (*n* = 39) conducted by Lindner et al., the presence of SARS-CoV-2 was identified in 61.5% of the analyzed myocardial samples, with signs of viral replication within the myocardial cells (in 15% of the analyzed samples), which was associated with premature death, compared to those without cardiac replication of SARS-CoV-2 [38,39]. Bearse et al. found even higher prevalence of SARS-CoV-2 in myocardial cells (73.17%, *n* = 30) of deceased COVID-19 patients, and this was associated with myocardial inflammation and ECG changes [40]. Case reports have related the presence of viral ribonucleic acid (RNA) in myocardial cells even without the involvement of the lung tissue in patients with fulminant myocarditis and cardiogenic shock [41,42].

The imbalance between the increased myocardial oxygen demand (due to the systemic hyperinflammatory status, fever, tachycardia, underlying cardiovascular disease, etc.) and the hypoxia due to respiratory failure can also lead to acute cardiac injury in COVID-19 patients. This condition may be further precipitated by underlying cardiac or metabolic disorders such as coronary artery disease, heart failure, diabetes mellitus, chronic kidney disease, etc. The hypercoagulable status due to the systemic inflammation, increased blood viscosity, vasospasm and the increase of shear stress can destabilize vulnerable coronary plaques and can subsequently lead to acute coronary syndromes. Microvascular thrombosis or pulmonary embolism can further aggravate hypoxia and the myocardial injury [8,24,43,44,45]. In a comprehensive review, Libby and Lüscher define COVID-19 as an “endothelial disease” given the fact that SARS-CoV-2 infection alters the endothelial homeostasis, which impairs the protective and nutritive function of the endothelium, thus generating multi-organ dysfunction and failure [46].

Infiltration of myocardium with inflammatory cells and the release of pro-inflammatory cytokines lead to increased oxidative stress, metabolic imbalance, acidosis and cell death with consecutive impairing of the ventricular function. The clinical manifestation varies from asymptomatic myocarditis with preserved left ventricular function to fulminant forms, with incessant arrhythmias, conduction blocks or cardiogenic shock. Basso et al. identified myocardial macrophage infiltration in 86% (18 out of 21) of the analyzed samples from COVID-19 patients, with only 14% (3 out of 21) showing histological signs of true myocarditis. There were no significant differences between the patients with myocarditis compared to those without myocarditis in terms of clinical features or cardiac troponin levels [47]. Current literature data reveal that myocardial injury is more common in COVID-19 compared to SARS-COV family infections, but the impact of each pathophysiological mechanism is yet to be determined [28].

Another possible clinical consequence of the cytokine storm is represented by stress-induced or takotsubo cardiomyopathy. The exact link is not yet well established, but recent retrospective studies evidenced an increased rate of takotsubo cardiomyopathy in critically ill COVID-19 patients, with severe inflammatory response syndrome [48]. Current hypotheses focus on both the increased catecholaminergic state during the disease, as well as the endothelial dysfunction and hyperinflammatory, pro-coagulant status in severe COVID-19 forms [49,50]. In a systematic review Sharma et al. noted that elevated pro-inflammatory cytokine (e.g., IL-6,-10, TNF-α) levels were associated with takotsubo cardiomyopathy and increased rates of adverse events and mortality [51].

## 4. COVID-19—A Trigger for Systemic Thrombosis

The signs of coagulation cascade activation (e.g., elevated serum D-dimer levels, thrombocytopenia, prothrombin time prolongation, fibrinogen) were noted in approximately half of patients from the Wuhan cohort and were associated with higher ICU admission rates and worse clinical outcomes [52,53,54]. Further pro-thrombotic biomarkers—such as the presence of lupus anticoagulant (LAC)—have been assessed in recent studies and reported the positive LAC in 45% to 91% in COVID-19 patients, specifically, in those with prolonged activated partial-thromboplastin time (aPTT) [55,56]. The presence of antiphospholipid antibodies has been reported in COVID-19 patients with arterial thrombosis complications, although the association with antiphospholipid syndrome has not been evidenced [57]. Moriarty et al. suggest that elevated levels of lipoprotein(a)—a validated risk factor for cardiovascular events—might also represent a thrombotic risk factor in COVID-19 patients [58,59].

Thrombotic events in hospitalized COVID-19 patients are relatively frequent complications with various clinical manifestations. These events can be attributed to coagulation disorders due to the hyperinflammatory status, hypoxia and lack of mobilization. In a Dutch cohort study, which included 184 severe COVID-19 patients admitted to the ICU, Klok et al. reported an incidence of 31% of thrombotic events, pulmonary embolism being the most frequent (81%), despite thromboprophylaxis [60]. Cantador et al. assessed the incidence of arterial thrombotic events in a large cohort study, which included 1419 hospitalized COVID-19 patients and reported that 1% (*n* = 14) of the study population suffered a systemic arterial thrombotic event, associated with 28.6% (*n* = 4) mortality rate. The most frequent arterial event was represented by stroke or transient ischemic attack (*n* = 8), followed by ACS (*n* = 3) and acute limb ischemia (*n* = 3). The events occurred mostly in the second week from onset of symptoms (8.5 ± 5.8 days) [61]. Several case report series evidenced massive carotid artery thrombosis in COVID-19 patients, with relatively low extent of atherosclerotic plaques and high levels of inflammatory biomarkers highlighting the impact of systemic inflammation and endothelial dysfunction on atherosclerotic plaques of any localization in the vascular system [62,63,64]. In a single center study, which included 388 COVID-19 patients from Milan, Italy, the authors reported a 7.7% (*n* = 28) cumulative incidence of arterial and venous thrombotic events, with higher rates of events in ICU patients compared to general ward (16.7%, 95%CI 8.7%–29.6% vs. 6.4%, 95%CI 4.2%–9.6%), with most frequent clinical presentation of venous thromboembolism (8.3% in ICU vs. 3.8% on general ward). Interestingly, 50% of the thromboembolic events were diagnosed in the first 24 h from admission, suggesting these events could be the first presentation of the disease and underlining the importance of active search for thrombosis in COVID-19 patients [65]. These findings are emphasized by the results of an autopsy study conducted by Wichmann et al. which evidenced venous thromboembolism in 58% (*n* = 7) of the autopsied bodies, with no suspicion of such events before the death of the patients. Furthermore, pulmonary embolism was the direct cause of death in 33% (*n* = 4) of the cases [66].

### 4.1. ACS and COVID-19—A Challenging Combination

A various range of clinical manifestations have been reported in COVID-19 patients presenting with an ACS, with frequent challenging scenarios for clinicians. In a case series report (*n* = 18) from New York, USA, Bangalore et al. reported a 33% prevalence of non-obstructive coronary artery disease (CAD) in COVID-19 patients with ST segment elevation who underwent coronary angiography for suspicion of a myocardial infarction. The overall in-hospital mortality was 72% with higher rates in patients with non-coronary myocardial injury (90%) compared to the myocardial infarction group (50%) [67]. In a study which included 28 patients with COVID-19, Stefanini et al. reported STEMI as the first manifestation of the disease in 85.7% (*n* = 24) of the study population. Non-obstructive CAD was identified in 39.3% (*n* = 11) of patients, with no identified cause for myocardial injury [68]. In an autopsy case report, Guagliumi et al. identified massive microvascular thrombosis in a COVID-19 patient with STEMI with patent epicardial arteries [69]. In an optical coherence tomography (OCT) controlled case report, Nakao et al. evidenced the presence of vasospasm and plaque erosion as the cause for STEMI in a COVID-19 patient with non-obstructive CAD [70]. A large thrombus burden has also been observed in COVID-19 patients with ACS, occurring with thrombosis in different vascular territories. Jenab et al. reported a case of simultaneous ACS, pulmonary embolism and cerebrovascular event in a COVID-19 patient, emphasizing the systemic nature of the disease with a hyperinflammatory and hypercoagulable status [71,72,73]. Another challenging clinical scenario—which can mimic an ACS—is represented by stress-induced or takotsubo cardiomyopathy. A rising incidence of takotsubo cardiomyopathy has also been reported during the COVID-19 pandemic. This increase has been attributed to both disease related factors (inflammation, catecholamine surge during the cytokine storm, microvascular dysfunction, etc.) and psychosocial stress factors, induced by the pandemic [74]. Recent systematic reviews evidenced that the presence of takotsubo cardiomyopathy in COVID-19 patients was associated with high complication rates (54–80%), irrespective of cardiac comorbidities and was more frequent in older female patients [51,52,53,54,55,56,57,58,59,60,61,62,63,64,65,66,67,68,69,70,71,72,73,74,75].

### 4.2. ACS Presentations in COVID-19 Era

Despite of the fact that COVID-19 favors plaque rupture and atherothrombotic events, an important decrease of ACS presentations has been reported worldwide. An Italian multicenter study compared the admissions for acute myocardial infarction (AMI) in the early period of 2020 lockdown with the similar period of 2019. A significant decline was noted in both ST elevation myocardial infarction (−26.5%, 95% CI: 21.7–32.3, *p* = 0.009) and non-ST-segment elevation myocardial infarction (−65.1%, 95% CI: 60.3–70.3, *p* < 0.001), with increased mortality rates in STEMI patients [4.1% vs. 13.7%, RR (95% CI) 3.3 (1.7–6.6)], *p* < 0.001] [76]. During the same period a substantial (58%) increase (362 vs. 229 cases) of out of hospital cardiac arrests were recorded compared to the same period of 2019 in the region of Lombardia, Italy, in a population of 1.5 million inhabitants [77]. Similar observations were published by Austrian authors, reporting a 39.4% decrease in ACS admissions during the course of 4-week observation period, resulting in an estimated 110 death surplus caused by ACS during the analyzed period [78]. Comparable findings were reported in multi center studies from the United States, with a 38–40.7% drop in admission for ACS compared to the similar period of 2019 [79,80]. These results are reflections of national lockdown strategies and the reluctance of the population to hospital admission because of the fear of contacting SARS-CoV-2.

## 5. Imaging Inflammation in COVID-19 Era

### 5.1. The Role of CMR for Detection of Cardiac Injury in COVID-19 Patients

Given the complex and hitherto not well-established cardiac involvement in the context of COVID-19 infection, there is a substantial need for detailed and accurate non-invasive assessment of the impact of the virus on cardiac structures and their prognostic relevance. CMR is the gold standard method for evaluation of cardiac chamber volumes, masses and function, along with dedicated tissue characterization sequences for differentiation between ischemic and non-ischemic cardiac lesions. These characteristics designate CMR as a valuable tool for evaluation of COVID-19 patients with cardiac injury and provide useful information for prompt differential diagnosis (myocarditis, myocardial infarction, takotsubo cardiomyopathy) and therapeutic decisions in challenging clinical scenarios [81,82]. Currently, there are several new CMR recommendations and scanning protocols that have been developed by imaging associations (e.g., European Association of Cardiovascular Imaging, Society for Cardiovascular Magnetic Resonance) for safe and efficient examinations in context of the COVID-19 pandemic [83,84].

**Figure 1:**
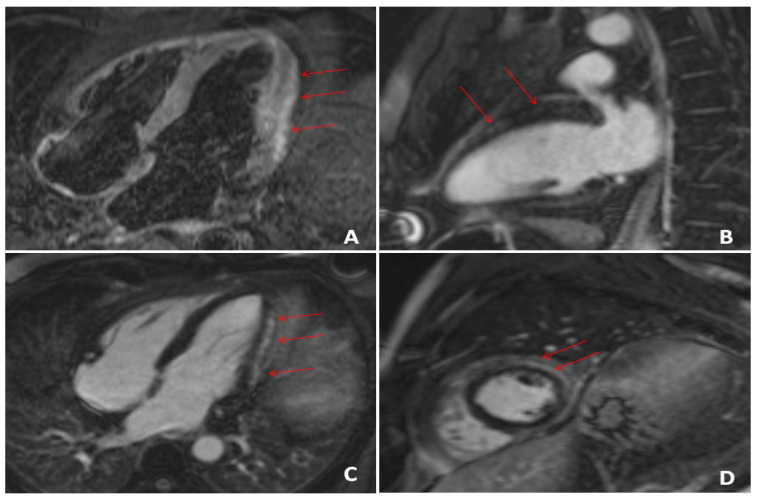
CMR assessment of a COVID-19 patient with elevated troponin levels. (**A**) Edema of the lateral wall in STIR sequences (arrows). (**B**) Non-ischemic subepicardial (arrows) LGE of the anterior wall in 2 chamber view; (**C**) 4 chamber view; (**D**) short axis (extended at 13% of the LV).

In the acute phase of the disease, short tau inversion recovery (STIR) sequences and T2 mapping techniques can identify the signs of myocardial inflammation (edema, vasodilation, hyperemia, pseudo hypertrophy) by detecting the free water content of the myocardial tissue. In the period of convalescence and the chronic phase, CMR can determine both morphological changes of the cardiac structure morphology, as well as the presence of irreversible myocardial fibrosis and necrosis in late gadolinium enhancement (LGE) sequences, which is an established prognostic factor for adverse cardiac events [11]. Advanced techniques, such as extracellular volume (ECV) and T1 mapping, which are capable of quantitative assessment of focal and diffuse fibrosis; edema; ventricular remodeling and the presence of thrombi, fat and iron in a highly accurate manner, which can further increase the diagnostic accuracy of CMR [85]. Figure 1 shows the CMR assessment of an acute phase COVID-19 patient with suspected myocardial involvement.

In recently published studies on COVID-19 patients, CMR proved to be efficient in detecting the etiology of cardiac injury evidenced by laboratory biomarkers (e.g., elevated troponin, MB fraction of creatine kinase), ECG changes or persistent symptomatology in lack of other causes (e.g., CAD) in every phase of the disease. In a case series report, which included 10 ACS suspected acute COVID-19 patients, Esposito et al. used CMR for elucidation of the diagnosis (following negative coronary angiography), which revealed myocardial inflammation (using STIR, T1 and T2 mapping) in 80% (*n* = 8) of the cases and 2 cases of takotsubo cardiomyopathy [86]. Other case reports evidenced prominent CMR abnormalities in the acute phase of COVID-19, which were characterized by diffuse myocardial edema detected in STIR and T1, T2 mapping sequences, with mild or no LGE, preserved LVEF and elevated serological biomarkers of inflammation and cardiac injury (e.g., CRP, troponin). Regarding the functional analysis, only mild or no left ventricular dysfunctions were noted in these reports, with no regional wall motion abnormalities [87,88,89,90]. These data suggest that myocardial edema (an indirect, validated hallmark of inflammation on CMR) may represent the only pathological finding in the initial stages of the disease. By identifying these early changes in the myocardial tissue, CMR is able to detect myocardial involvement before the appearance of wall motion abnormalities. The general features of the main studies using CMR in COVID-19 are summarized in Table 1 and CMR characteristics are listed in Table 2.

In a CMR follow-up study, published by Wang et al., which included 44 patients recovered from COVID-19, the authors found evidence of myocardial injury in 30% of the cases, defined by myocardial or sub-epicardial LGE, with no signs of edema at 3 months. Furthermore, the LV functional analysis revealed significantly lower peak global circumferential strain (GCS) rates in LGE positive subjects compared to healthy controls (−15.1 ± 10.3 vs. −19.4 ± 3.0, *p* = 0.04). The RV peak GCS (−9.4 ± 3.4 vs. −12.1 ± 4.0, *p* = 0.05) and global longitudinal strain (GLS) rates (−7.8 ± 4.0 vs. −12.9 ± 3.0, *p* = 0.003) were also significantly lower in the LGE positive group compared to the LGE negative group [97]. In the largest published CMR study to date on convalescent COVID-19 patients, which included 148 recovered from severe form of the disease with positive troponin, Kotecha et al. reported CMR abnormalities in 54% (*n* = 80) of the patients, with myocarditis-like pattern in 27% (*n* = 40), with 30% of these showing signs of active myocarditis at a median of 68 days. LGE was present in 49% (*n* = 70) of the patients with most frequent localization in the subepicardial region 19% (*n* = 28). The LV function was preserved in 89% of the included patients, concluding that myocarditis related functional aftermaths are limited. The authors reported evidence of ischemia in 28% (*n* = 41) of the subjects with presence of myocardial infarction in 19% (*n* = 28). From the ischemic CMR pattern group, 66% (*n* = 27) were not known with history of CAD [95]. In a previous study that included 100 recovered COVID-19 patients, Puntmann et al. reported CMR abnormalities in 78% of the subjects, which were independent of the severity of the disease. In addition, 60% of the patients evidenced CMR signs of active inflammation (native T1, T2 abnormalities) at a median of 68 days from the initial diagnosis [96]. In a recent systematic review, Ojha et al. analyzed the CMR features of 199 COVID-19 patients from 34 studies. The authors concluded that only 21% of the CMR results were without pathological findings, and the most frequent CMR diagnosis was represented by myocarditis (40%, *n* = 80). Two thirds of the patients evidenced T1/T2 abnormalities with CMR signs of edema, and LGE was present in 43% (*n* = 85), with mostly (81%) subepicardial localization. The LV function was preserved in most of the cases [98].

### 5.2. CTA and Multimodal Imaging in COVID-19 Patients

**Figure 2:**
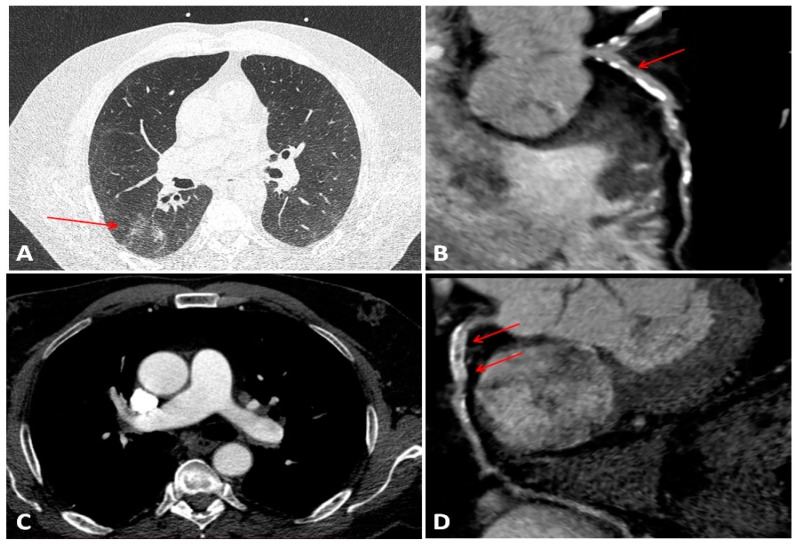
CT assessment of a patient presenting with rapid onset dyspnea with positive D-dimer test and COVID-19 PCR swab. (**A**) “ground glass” opacities on the right posterior basal segments (arrow); (**B**) mixed (arrow) and calcified atherosclerotic plaques with severe stenosis on the left anterior descending artery (LAD); (**C**) permeable pulmonary arteries; (**D**) stent on the proximal right coronary artery with moderate in-stent restenosis (arrow).

Chest CT imaging is a cornerstone for evaluation of the extent of the lung involvement in COVID-19 patients. Current post-processing techniques facilitate the extraction of various type of date from conventional CT images. These methods permit the assessment of lung parenchyma, coronary artery disease, pulmonary embolism or local vascular inflammation and even wall motion abnormalities. Complex CT evaluation of a confirmed COVID-19 patient are shown in Figure 2. A new non-invasive imaging biomarker has been proposed by Antonopoulos et al. for the assessment local vascular inflammation by determining the perivascular fat attenuation index (FAI). It is based on measurement of PVAT CT gradient by determining the transcriptomic profile (phenotypic changes of PVAT bordering coronary plaques) with the use of artificial intelligence (AI). FAI exhibited high sensitivity and specificity in detection of inflammation, validated by 18F-fluorodeoxyglucose uptake in positron emission tomography (PET-CT) [99]. In an Italian registry study which included 1625 patients, Scoccia et al. evaluated the prognostic value of coronary calcium score (CAC) from non-gated chest CT in patients hospitalized for COVID-19. The study concluded that the burden of CAC was associated with higher in-hospital mortality rates and both cardiac and cerebrovascular events. Furthermore, CAC was identified as a superior prognostic marker compared to clinical cardiovascular risk assessment [100]. In addition, the registry investigators added that total thoracic calcium (which consists of CAC, thoracic aorta calcium and aortic valve calcium) possesses an even better prognostic value for mortality in comparison with CAC [101]. In a multi-center cohort study, Esposito et al. identified that an increased diameter (>31 mm) of the main pulmonary artery was an independent predictor for mortality in hospitalized COVID-19 patients [102].

Considering the multi-organ involvement of COVID-19, there is no single imaging method that can provide the necessary information for proper diagnosis and management of these complex cases. In the epidemiological context of the pandemic, the choice of the imaging modality is cumbersome due to the need of specific personal protective equipment, limitation of personal contact with the infected patients and efficient disinfection of the rooms and devices. Several protocols and recommendations have been proposed for safe and efficient multimodal cardiovascular imaging in setting of the pandemic [103,104]. Citro et al. proposed a diagnostic algorithm for evaluation of COVID-19 patients with elevated troponin levels. The initial evaluation is based on the presence of ST elevation on ECG, followed by echocardiography with identification of ischemic or non-ischemic wall motion abnormalities. CT angiography is proposed for triple rule out of pneumonia, pulmonary embolism and CAD in patients without ST segment elevation and no ischemic wall motion abnormalities, completed by CMR with functional and tissue characterization assessment [105]. In addition, Pontone et al. highlighted the possibility of quadruple rule-out (pneumonia, pulmonary embolism, CAD and myocarditis) using iodine-enhanced CT scan protocols [106]. Rudski et al. developed a complex algorithm for the use of multimodal imaging methods of cardiovascular complications of COVID-19 patients, based on the phase (acute, convalescent and chronic), the clinical scenario (chest pain, suspected ACS, new LV dysfunction) and severity (hemodynamic stability/instability) of the disease [107].

## 6. Future Perspectives—The Role of Artificial Intelligence in COVID-19 Patients

**Figure 3:**
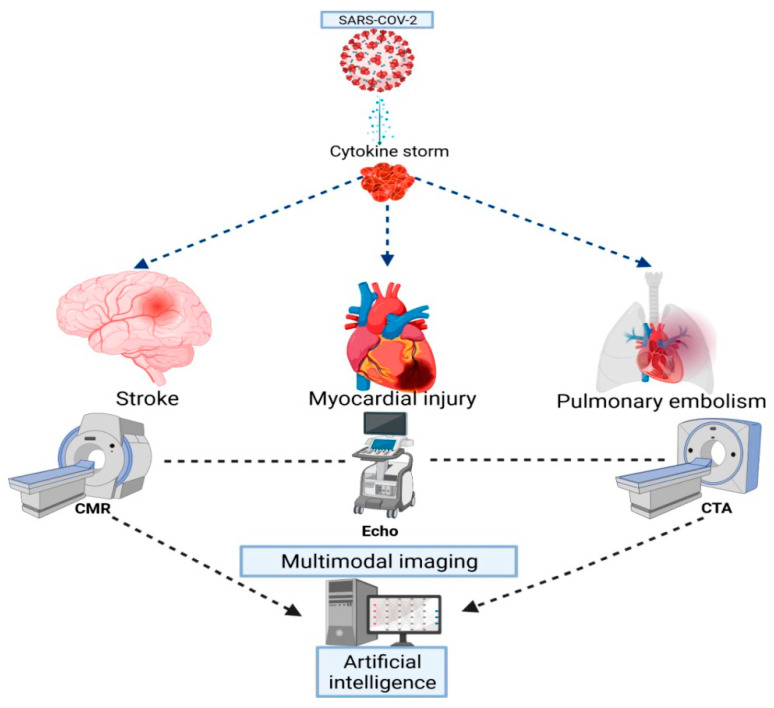
Summarization of cardiovascular imaging techniques in COVID-19 patients.

Artificial intelligence (AI) and machine learning algorithms have been already used in cardiovascular medicine for imaging based cardiovascular risk assessment [108,109,110]. The rapidly accumulating and broadly various data acquired from COVID-19 studies overburden medical professionals in finding the most efficient diagnostic and therapeutic methods. From the early days of the pandemic, AI has been used for identifying the virus structure, spreading and possible therapeutic approaches [111,112]. Several studies have demonstrated the capability of AI in automatic differentiation of COVID-19 pneumonia from other form of pneumonia based on chest X-ray and CT images [113,114]. Kang et al. reported a 91.2% accuracy and 95% severity assessment of COVID-19 and bacterial pneumonia using qualitative AI analysis [115]. Suri et al. assessed the role of AI in identifying myocardial and brain damage in COVID-19 patients based on available clinical and imaging data (echocardiography, X-ray, CT, CMR scans). The authors concluded that AI algorithms were able to provide risk stratification of patients, ranging from “no risk” to “high-high” risk and were also able to develop diagnostic pathways with recommendations for appropriate imaging method for every clinical scenario [116]. Figure 3 summarizes the application of cardiovascular imaging techniques for evidencing inflammation and cardiovascular complications in COVID-19 patients.

## 7. Conclusions

The inflammatory response associated with COVID-19 infection mainly mediates the course of the disease, with multi-organ involvement. Early diagnosis and treatment of systemic or local inflammatory reaction is the key of avoiding severe forms. Cardiovascular involvement is one of the most frequently met complications in COVID-19 and detection of cardiac injury via efficient imaging methods may help to avoid unnecessary investigations and to provide optimal treatment in each phase of the disease. The use of AI can further improve the diagnostic accuracy of these imaging techniques providing risk stratification and better prognostic value.

## Figures and Tables

**Table 1:** General characteristics of the main studies on cardiac involvement in COVID-19.

Reference	COVID-19 Phase	Study Type	Nr. of Patients	Age	Cardiological History	Clinical Presentation	Pulmonary Findings
Esposito et al. [86]	Acute (1 week)	Case reports	10	52 ± 6	No	Chest pain, dyspnea	NA
Gravinay et al. [87]	Acute (8 days)	Case report	1	51	NA	Atypical chest pain, dyspnea, fever, arthromyalgia	None
							
Luetkens et al. [88]	Acute (10 days)	Case report	1	79	No	Dyspnea, syncope, fatigue	Ground glass infiltrates
							
Manka et al. [89]	Acute (Day 5)	Case report	1	75	HTN, obesity, CKD	Fever, chills, cough, dyspnea	Pneumonia
							
Caballeros et al. [90]	Acute	Case reports	2	26, 13	Gestational diabetes	Chest pain, fever, cough	None
							
Rajpal et al. [91]	Early convalescence (11–53 days)	Research letter	26	19.5	NA	26.9% mild symptoms 73.1% asymptomatic	NA
							
Knight et al. [92]	Early convalescence (46 ± 15 days)	Retrospective observational	29	64 ± 9	NA	NA	69% with residual lung parenchymal changes
							
Huang et al. [93]	Early recovery 47 (36–58)	Retrospective observational	26	38 (32–45)	8% HTN	Chest pain, palpitations, chest distress	NA
							
Ng et al. [94]	Convalescence	Retrospective observational	16	68 (53–69)	6.25% CAD	94% mild/moderate	NA
							
Kotecha et al. [95]	Convalescence (median 68 days)	Prospective observational	148	64 ± 12	57% HTN 46% Hypercholesterolemia 34% DM	Sever COVID-19 at presentation, 32% with ventilatory support	NA
Puntmann et al. [96]	Convalescence 71 (64–92 days)	Prospective observational	100	49 (45–53)	22% HTN 18% DM 22% Hypercholesterolemia 13% CAD	Chest pain, palpitation, shortness of breath	NA
Wang et al. [97]	Mid-term recovery (102.5 ± 20.6 days)	Prospective observational	44	47.6 ± 13	25% HTN 18.2% DM 36.4% Hyperlipidemia 4.6% Hepatitis B	NA	NA
							

HTN—Hypertension, CAD—coronary artery disease, DM—diabetes mellitus, CKD—chronic kidney disease, NA—not assessed.

**Table 2:** CMR findings of the main studies on myocardial involvement in COVID-19.

Reference	Diagnosis	Edema	LGE	ECV	LVEF	WMA	Perfusion Deficit	Other
Gravinay et al. [87]	Myocarditis	Subepicardial	+	NA	Preserved	None	NA	LV thrombus
Esposito et al. [86]	Myocarditis Takotsubo	Diffuse (STIR, T1, T2 mapping)	+ 1–3% of LV (*n* = 3)	*n* = 2 (30, 36%)	<40% (*n* = 2) 40–55% (*n* = 3) >55% (*n* = 5)	NA	NA	Pericardial effusion
								
Luetkens et al. [88]	Myocarditis	Diffuse (T1, T2 mapping)	-	NA	49%	Global hypokinesis	NA	Pericardial effusion
								
Manka et al. [89]	Acute myocardial injury	Global (STIR, T1, T2 mapping)	-	NA	59%	No	NA	-
Caballeros et al. [90]	Myocarditis	Basal/inferior septum T1, T2 mapping)	+ (14.2% of LV mass)	NA	59%	NA	NA	-
	Myocarditis	Ventricular septum (T2, native T1)	-	NA	Normal	NA	NA	Pericardial effusion
Rajpal et al. [91]	15% Myocarditis	15% (elevated T2)	+ 46% (*n* = 12)	Elevated in 3.8% (*n* = 1)	58.6%	NA	NA	7.5% % with pericardial effusion
								
Knight et al. [92]	44% Myocarditis 31% Ischemic	No	38% non-ischemic 17% ischemic 14% dual	NA	67.7% ± 11.4	NA	Done in 66% (*n* = 19) 47% ischemia 42% inducible ischemia	7% with pericardial effusion 14% pleural effusions
								
Huang et al. [93]	NA	+ 54% (*n* = 14)	+ 31% (*n* = 8)	28.2%	60.7% ± 6.4	NA	NA	Pericardial effusion
								
Ng et al. [94]	19% Myocarditis	25% elevated native T1 25% T1 and T2 5% native T2	+ 25%	NA	59% (56–65)	NA	NA	NA
Kotecha et al. [95]	27% Myocarditis 19% MI	30% of the myocarditis pattern patients	+ 49% (*n* = 70)	NA	67% ± 11	No regional WMA in myocarditis pattern	Done in 51% (*n* = 76) 28% IHD	5% with pericardial effusion 6% pleural effusions
Puntmann et al. [96]	78% Myocarditis	60% with abnormal native T2	+ 20% non-ischemic pattern 12% ischemic pattern	NA	56% (54–58)	NA	NA	22% pericardial LGE 20% pericardial effusion
Wang et al. [97]	Myocardial injury	NA	29.5% Mid-myocardial Sub-epicardial	NA	64.3% ± 5.9 in LGE+ 62.2% ± 4.4 in LGE−	RV peak GCS −9.4 ± 3.4(LGE+) vs.−12.1 ± 4.0 (LGE−) *p* = 0.04	NA	NA

LGE—late gadolinium enhancement, ECV—extracellular volume, LV—left ventricle, LVEF—left ventricle ejection fraction, WMA—wall motion abnormalities, RV—right ventricle, GCS—global circumferential strain, IHD—ischemic heart disease, STIR—short tau inversion recovery, NA—not assessed, + present.

## Data Availability

Not applicable.
